# Group-level component analyses of EEG: validation and evaluation

**DOI:** 10.3389/fnins.2015.00254

**Published:** 2015-07-29

**Authors:** Rene J. Huster, Sergey M. Plis, Vince D. Calhoun

**Affiliations:** ^1^Department of Psychology, University of OsloOslo, Norway; ^2^The Mind Research NetworkAlbuquerque, NM, USA; ^3^Department of Computer Science, University of New MexicoAlbuquerque, NM, USA; ^4^Department of Electrical and Computer Engineering, University of New MexicoAlbuquerque, NM, USA

**Keywords:** EEG, group component analysis, multisubject analysis, blind source separation, ICA, SOBI

## Abstract

Multi-subject or group-level component analysis provides a data-driven approach to study properties of brain networks. Algorithms for group-level data decomposition of functional magnetic resonance imaging data have been brought forward more than a decade ago and have significantly matured since. Similar applications for electroencephalographic data are at a comparatively early stage of development though, and their sensitivity to topographic variability of the electroencephalogram or loose time-locking of neuronal responses has not yet been assessed. This study investigates the performance of independent component analysis (ICA) and second order blind source identification (SOBI) for data decomposition, and their combination with either temporal or spatial concatenation of data sets, for multi-subject analyses of electroencephalographic data. Analyses of simulated sources with different spatial, frequency, and time-locking profiles, revealed that temporal concatenation of data sets with either ICA or SOBI served well to reconstruct sources with both strict and loose time-locking, whereas performance decreased in the presence of topographical variability. The opposite pattern was found with a spatial concatenation of subject-specific data sets. This study proofs that procedures for group-level decomposition of electroencephalographic data can be considered valid and promising approaches to infer the latent structure of multi-subject data sets. Yet, specific implementations need further adaptations to optimally address sources of inter-subject and inter-trial variance commonly found in EEG recordings.

## 1. Introduction

Recent years saw a rapid advance in the development of methods for the analysis of large multi-subject data sets in neuroscience, not least because signals and images obtained from the brain are complex and hard to structure without reverting to computational methods (Lemm et al., 2011). A predominant goal of current developments is to directly make inferences on the general nature or structure of neurocognitive processes underlying a specific task context or disease state. Many of these group-level analyses have been developed in the context of functional magnetic resonance imaging (fMRI, e.g., Calhoun and Adal, 2012), but more recent developments also address other modalities such as electroencephalography (EEG). However, each modality comes with its own peculiarities that need to be addressed. This paper sets out to evaluate applications of independent component analysis (ICA) and second order blind identification (SOBI) for the analysis of multi-subject EEG data, focusing on approaches that directly infer a structure of sources common to subjects and considering major sources of variance in EEG, namely temporal jittering of neuronal responses and inter-individual variability of scalp topographies.

**Figure 1:**
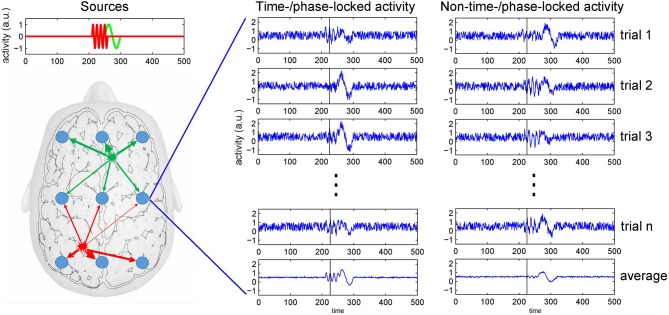
**The mixing problem of EEG and trial averaging**. Neuronal activity is picked up at scalp electrodes (blue circles), with recordings at each electrode originating from a specific mixture of brain sources (as indicated by the arrows' thickness). To the right, simulated recordings from an electrode are shown. The left of the two columns depicts source activities that are time- or phase-locked to an event. The right column depicts onset times that show some temporal variability across trials. Because at the same point in time (see black bar) the activity can be in different phases of the neuronal response (e.g., either at a peak or a trough), averaging only restores a distorted version of the source activities as recorded at an electrode.

EEG is acknowledged as an important tool to study perceptual processing and cognition as it measures neuronal activity reflected in spatio-temporal patterns of voltage fluctuations across a subject's scalp (Nunez and Srinivasan, 2006; Buzski et al., 2012). With EEG, the spatially and temporally summed current flows resulting from synaptic activity at the neurons' dendrites are considered the major source of voltage fluctuations registered at the head's surface. To be detected at an EEG electrode, currents generated at a specific brain region have to traverse through the different tissue types of the brain, as well as the scull and the scalp. This process usually is referred to as volume conduction, which causes surface electrode recordings to reflect mixtures of the temporal profiles of concurrently active brain sources (Nunez et al., 1997; Winter et al., 2007). Hence, a specific event recorded in the EEG most likely does not reflect a single process or activity of a single brain region. Figure 1 depicts the mixing of brain sources at recording electrodes.

Because of this mixing problem, blind source separation via ICA for EEG has become increasingly popular (Hyvrinen and Oja, 2000; Onton et al., 2006). When applied to EEG data, ICA reconstructs source activity time courses by maximizing their statistical independence while estimating the parameters of the underlying (de-)mixing process. The most common application of ICA to EEG without a doubt is artifact correction: ICA is used to estimate and deplete the activity of artifactural sources (e.g., the activity of facial muscles or eye blinks; e.g., Hoffmann and Falkenstein, 2008), in the end reconstructing artifact-free EEG. On the other hand, ICA also is used to isolate and extract desired brain activity. Hence, by demixing the original EEG recordings, ICA helps to identify and separate the activity of single brain sources, thereby also increasing the SNR of the reconstructed EEG events of interest (Delorme et al., 2012). In the most common applications, though, ICA is applied to data of single subjects and therefore does not naturally generalize to a group of subjects, because the number of sources or their exact constellation may well differ across a pool of subjects. That is, even when working on the same task, subjects may use different cognitive strategies thereby causing variations with regard to the number of active brain sources or their mixing. In addition, no two brains are identical and because EEG recordings rely on volume conduction, differences in brain anatomy alone can cause variations in the sources' mixing as found at surface electrodes (Michel et al., 2004).

Another source of variance is a potential variability of the onset of neuronal processes in response to external or internal events. The computation of an event-related potential (ERP) rests on the assumption that the triggered neuronal processes show no variability regarding temporal onset and peak. However, so-called induced responses show a substantial temporal jittering such that simple averaging over trials does not only reduce noise, but also reproduces a significantly depleted or distorted version of the sources' signals only (Makeig et al., 2004). Thus, when (pre-)processing EEG data care has to be taken not to lose significant portions of the signal that might be of interest in a given task or analysis context (see Figure 1).

To allow for inferences about the source configuration at the group-level using ICA, two strategies have been suggested. First, single-subject ICAs are computed and the components are matched across subjects using clustering algorithms (e.g., Bigdely-Shamlo et al., 2013). Second, components constantly expressed across subjects can be estimated using a single ICA computed on aggregate data sets built from EEG recordings of multiple subjects. Algorithms of the latter class usually are referred to as group ICA (e.g., Eichele et al., 2011; Cong et al., 2013). These two approaches can be considered as opposites on a methodological spectrum. The clustering of single-subject components holds the potential problem that many of the resulting clusters will be sparse, i.e., not all subjects contribute to all clusters. Whereas one might argue that this corresponds to a true representation of the latent sources, one cannot easily make statistical inferences at the group level anymore, since subjects may show extremely unequal contributions to many clusters. Hence, this approach focuses on the integrity of an individual's representation at the expense of limited capabilities for population-level inferences. Group ICA, on the other hand, aims at extracting components consistently expressed across subjects and has extensively been used for the identification of networks in fMRI (Calhoun and Adal, 2012). Since a set of components is estimated that is common to all subjects, this model naturally translates to population-level inferences and can easily be applied to group comparisons. Two approaches for group ICA have been suggested and their evaluation will be the focus of this study.

**Figure 2:**
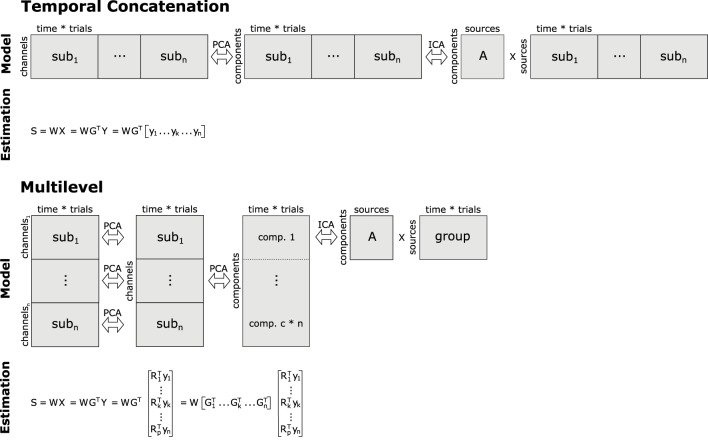
**Basic concepts of group analyses using temporal concatenation and multilevel decomposition in combination with ICA or SOBI**. For temporal concatenation, data aggregation yields a horizontally elongated matrix on which the demixing matrix *W* can be estimated, assuming the same mixing process for all subjects. This, however, is not the case with multilevel decomposition since single-subject as well as group-level decomposition prior to ICA/SOBI not only reduce the number of variables, but also allow for some variability of the latent structure across subjects. Note that usually only a subset of the c*n vertically concatenated components (c = number of channels/components, n = number of subjects) enter final decomposition via ICA or SOBI.

Computing a group ICA simply by temporally concatenating the data of multiple subjects has already been suggested about a decade ago (Delorme and Makeig, 2004), but was only recently published in a more formal framework (Cong et al., 2013). In this approach, an aggregate data set is built from matrices containing the single-subject EEG data. Specifically, let *y*_*k*_ be the EEG data of the *k*-th subject containing EEG recordings from *m* electrodes as row elements, and *o* samples as columns. Then, the aggregate data set *Y*^*h*^ of size *m* × (*n* * *o*) is given by the horizontal (temporal) concatenation $Y^{h}=\left[y_{1},\ldots ,y_{k},\ldots ,y_{n}\right]$, with *k* = 1, …, *n*, and *n* being the total number of participants included in the analysis. Furthermore, we apply centering and whitening via principal component analysis (PCA) to *Y*^*h*^, providing us with the principal components *R*^*T*^*Y*^*h*^, where *R* corresponds to the orthonormal transformation matrix obtained from PCA^1^. Applying the basic ICA model to the preprocessed data leads to *R*^*T*^*Y*^*h*^ = *AS*^*h*^, where $S^{h}=\left[s_{1},\ldots ,s_{k},\ldots ,s_{n}\right]$ is the matrix containing the horizontally/temporally concatenated component time courses (estimated source activities) of the *n* participants. Note, that this design implies that *A* = *A*_1_ = … = *A*_*k*_ = … = *A*_*n*_, i.e., the mixing matrices of the different subjects are identical. Hence, the number of sources and their variances must not vary across the single-subject data sets. Because this approach to group ICA of EEG relies on temporal concatenation of single-subject data sets, we will refer to it as tcICA. Please refer to Figure 2 for a depiction of this approach.

Following the notion that different participants or separate conditions may produce differing sets and constellations of components, Eichele et al. (2011) proposed a different approach to group ICA of EEG. Their procedure encompasses two consecutive data reduction steps with subsequent reorganization and aggregation prior to ICA (see Figure 2). Specifically, let *y*_*k*_ again be an EEG data set of subject *k* with *m* electrodes. In the first step, for each of the *n* subjects a PCA is computed on their data such that we receive the subject-specific principal components $R_{k}^{T}y_{k}$, where $R_{k}^{T}$ is the transposed *c*_1_ × *m* PCA-transformation matrix, where *c*_1_ corresponds to the number of principal components that have been extracted at this level. Note that both $R_{k}^{T}$ and *y*_*k*_ are specific for the *k*-th subject. Then, an aggregate data set of size (*n* * *c*_1_) × *o* is built via vertical (spatial) concatenation of the *n* reduced data sets $R_{k}^{T}y_{k}$. Note that *c*_1_ usually is smaller than *m*, because PCA often is used for data reduction. On this aggregate data set a second PCA is computed, now extracting *c*_2_ group principal components. At this step, we receive matrix *X*, which is of size *c*_2_ × *o* and contains the group principal components computed via matrix *G*^*T*^, which is the transformation matrix of the group PCA. Note that in the basic application of PCA both $R_{k}^{T}$ and *G*^*T*^ would be square, whereas here these matrices may have been reduced to *c*_1_ and *c*_2_ rows, respectively. Finally, we can compute the *c*_2_ group independent components from the group principal components according to *S* = *WX*, where *S* is the *c*_2_ × *o* matrix with the group independent component time courses, and *W* is the *c*_2_ × *c*_2_ demixing matrix of the group ICA.

In contrast to tcICA, this model does not directly deliver subject-specific time courses of the independent components, but rather some aggregate, group independent component time courses. However, subject-specific independent component time courses *s*_*k*_ can easily be computed via multiplication of the original data, the subject-specific elements of the first- and second-level PCAs, as well as the demixing matrix: $s_{k}=WG_{k}^{T}R_{k}^{T}y_{k}$. Similarly, the subject-specific topographies of an independent component can be reconstructed by $t_{k}=A^{T}G_{k}^{T}R_{k}^{T}$, where each row of *t*_*k*_ contains the weights indicating a component's contribution to a given EEG channel. Because the approach of Eichele et al. (2011) relies on nested component analyses computed at multiple levels, we will refer to it as mlICA.

It can clearly be seen that these two approaches, tcICA and mlICA, should exhibit different strengths and weaknesses with regard to the above-mentioned sources of variance in EEG. With tcICA it is assumed that the mixing of sources, and correspondingly their scalp topographies, do not differ across subjects. It seems reasonable, however, that this assumption will be violated in nearly all multi-subject EEG data sets to some extent. Therefore, Cong et al. (2013) suggested to first assess the model order of the subject-specific data sets, for example by using the Akaike or Bayesian information criteria, then testing whether there is sufficient homogeneity of these indices to warrant the application of tcICA (Cong et al., 2013). Note though, that the absence of inter-individual variation in terms of the number of sources is a necessary criterion for this group ICA approach, but not a sufficient one. Two subjects may show the same number of sources, but the individual mixing matrices may still differ, simply because corresponding sources may project differently to the scalp or because the spatial, temporal, or frequency characteristics of sources do not match across subjects (i.e., a specific source is actually not expressed in every subject). With mlICA, on the other hand and although only a single common demixing matrix *W* is estimated for all subjects, inter-subject variability in the weighting of independent components to the single-subject scalp recordings is allowed. This is because not only the single-subject PCA, but also the group PCA may show inter-individual variation in their transformation matrices. Note that the transformation matrix *G*^*T*^, as obtained in the context of mlICA (but not tcICA), can be partitioned into subject-specific $G_{k}^{T}$, thereby providing the contribution of the subject-specific principal components to the group principal components, and ultimately also the group independent compenents. This compensates for differences regarding the number or nature of sources across subjects. However, when considering temporal jittering of source activity, mlICA should exhibit a bias toward activity patterns consistently expressed and correlated across subjects, since the group PCA will select for such activity patterns and non-consistent activity might be lost during this data reduction step. Temporal jitter should not lead to deteriorated performance with tcICA, though, as long as the mixing matrices indeed show a high degree of similarity. This is because volume conduction will still guarantee high correlations across channels and tcICA's PCA only considers correlations across channels but not subjects.

Infomax ICA (Bell and Sejnowski, 1995) has shown good performance for the analysis of neuroscientific data and usually is the algorithm of choice also in applications of tcICA and mlICA. Recently it has been argued, though, that algorithms relying on second order statistics may be more adequate to use when variations in source mixing are to be expected. Lio and Boulinguez (2013), for example, showed that SOBI (second-order blind identification; Belouchrani et al., 1997) performs significantly better than Infomax ICA in context of group ICA for EEG (using temporal concatenation), when mixing matrices vary slightly as it would result from differences in electrode placements. It still needs to be determined though, whether this finding still holds with more pronounced differences in mixing matrices. Hence, within the framework of this study two additional applications for the estimation of group components, namely tcSOBI and mlSOBI, are derived by replacing ICA with SOBI.

With this study, we will put temporal concatenation as well as the multilevel data decomposition to test under controlled yet realistic conditions, using both Infomax ICA and SOBI as algorithms for blind source separation. Multi-subject EEG data will be simulated using a known set of sources whose activity profiles vary in terms of frequency, phase-locking, as well as their topographical mapping. In an initial simulation the generated signals will be embedded in random Gaussian noise, whereas a second simulation will use real EEG measurements of subjects at rest as background signal. Note that the former case is a well-controlled state, because the number of relevant sources as well as their contributions to the surface EEG are specified. When using real resting state EEG as noise, however, we have to deal with an additional and unspecified number of distractor sources whose strengths are not known. Ultimately, this study will provide initial guidelines for the application of group-level ICA and SOBI for EEG, as well as important reference points for further development.

## 2. Materials and methods

### 2.1. Design

To evaluate the performance of these algorithms under realistic conditions, multi-subject EEG data were simulated roughly mimicking brain responses as observed in a simple response task with visual stimulation. A total of five factors were varied. (I) Three sources were modeled expressing activity relative to a virtual event. One source was characterized by an early 40 Hz gamma band response showing largest activity at occipital electrodes over the visual cortex. The second source modeled increased activity over medial electrodes in the alpha band with a spectral peak at 10 Hz. The third source reflected beta band activity at 20 Hz over the left motor cortex, modeling EEG activity associated with a button press. The amplitudes of the response profiles of the three sources were varied independently from trial to trial. (II) The single-trial onset times of the sources were varied in five steps ranging from a jitter of 0–200 ms around an average onset time, thus modeling a transition from perfectly time- or phase-locked (evoked) to non-phase locked (induced) brain activity. Specifically, onset times could vary up to a total of either 0, 25, 50, 100, or 200 ms. (III) Topographies resulting from a given source could either be constant or variable across subjects; i.e., the single-subject mixing matrices were either the same or varied. (IV) The two approaches for group analysis and their combinations with ICA and SOBI (tcICA, mlICA, tcSOBI, mlSOBI) were compared. (V) The simulated EEG was embedded into background noise. This background noise was either artificially generated (Gaussian random noise) or taken from real EEG recordings of participants at rest.

### 2.2. Simulations

**Figure 3:**
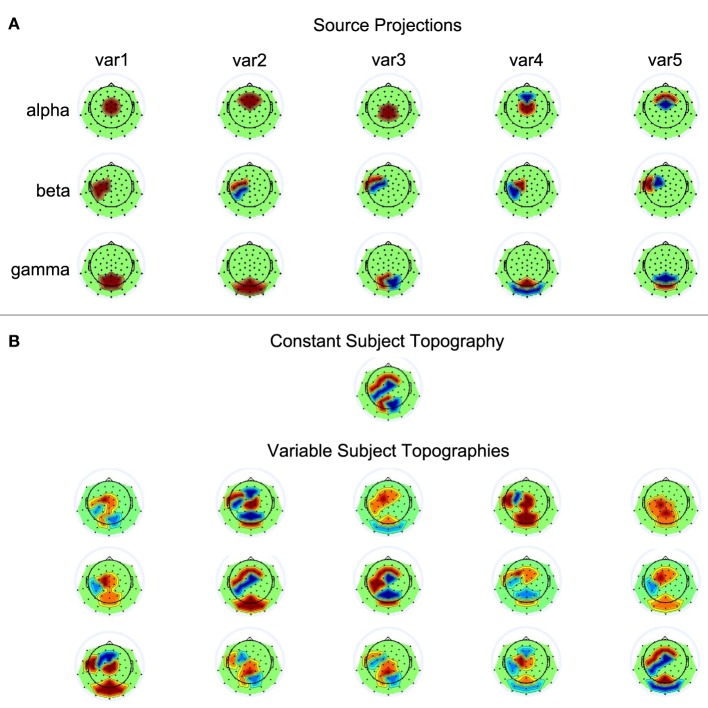
**Source projections**. **(A)** The first, second, and third row each contain the five scalp projections used for the alpha, beta, and gamma source, respectively. Black dots on the scalp indicate electrode positions. Red and blue represent positive and negative weights, respectively. **(B)** Shown are the simulated subject topogrpahies that could either be constant or variable across subjects.

Fifteen single-subject data sets were simulated, each containing data from 62 electrodes and 50 trials. Each trial contained three seconds of data generated at a sampling rate of 500 Hz. The three sources showed activity relative to a virtual event occurring at 1000 ms. The three sources showed sinusoidal activity of 100 ms duration at 10 Hz (alpha), 20 Hz (beta), and 40 Hz (gamma), with average onset times of 400, 600, and 200 ms post stimulus presentation, respectively. Exact onset times of the sources could vary across trials in accordance with five conditions: time windows for temporal jittering were centered at the previously described average onset times and allowed source onset variability of 0, 25, 50, 100, or 200 ms. The average amplitude of a source was scaled such that it corresponds to an average surface EEG amplitude of 10 µV. Across trials, however, source amplitudes varied between 5 and 15 µV. The exact values for amplitude and latency variations were generated via random selections from a uniform distribution. To transform source activities to scalp EEG, five varying projections were defined for each of the sources. That is, the projection vector defined a source's contribution to each of the electrodes by setting the corresponding loading to 0, 1, or −1. The EEG can be simulated by computing *Y*_*sim*_ = *ÂS*, where *Â* is the sparse 62 × 3 mixing matrix of our model with columns containing the projection vectors of the sources, and *S* the 3 × (1500 × 50) matrix with the source time courses of the temporally concatenated trials. The varying projection patterns for the three sources are shown in Figure 3A. The single-subject data sets were simulated according to two conditions, with *Â* either being constant or different across subjects. To generate variable topographies according to the latter condition, for a given subject the source projections were randomly chosen from the set of possible assignments displayed in Figure 3A, with the additional constraint that no two mixing matrices must be identical. Please refer to Figure 3B for a depiction of the resulting source-to-scalp projections.

At last, noise was added to finalize the generation of the simulated data. In one condition, noise was generated by selecting random numbers from a Gaussian distribution. In a second condition excerpts from real EEG measurements of 15 subjects at rest (eyes open and focusing on a fixation cross) were taken as approximations of real EEG noise and added to the simulated data. The Gaussian noise was generated such that it matched basic characteristics of the EEG noise; hence, noise under both conditions exhibited a mean of 0 and a standard deviation of 8.5. The EEG recordings correspond to calibration measurements of the first session of a neurofeedback training study (Enriquez-Geppert et al., 2014). This study was approved by the ethics committee of the University of Oldenburg, Germany.

The simulated data sets were subjected to group analyses as discussed in the Introduction. In case of tcICA and tcSOBI, data were horizontally concatenated to form the multi-subject data set $Y_{sim}^{h}$. This multi-subject data set was centered and whitened using PCA. However, PCA was not used to reduce data dimensionality; rather, all 62 principal components were then subjected to Infomax ICA or SOBI. Finally, this provides us with data matrix ${\tilde{S}}_{sim}^{h}=\left[{\tilde{s}}_{1},\ldots ,{\tilde{s}}_{k},\ldots ,{\tilde{s}}_{n}\right]$ that contains the horizontally/temporally concatenated components or source estimates of the *n* participants. These subject-specific component time courses were later used for the evaluation of the algorithms' performance.

With mlICA and mlSOBI, first, all single-subject data sets separately undergo centering and whitening via PCA, again using all 62 principal components for further processing. Then, a multi-subject data set is built by vertically concatenating the single-subject principal components and a second PCA is computed on these data. Now, the first 62 group principal components, i.e., those contributing most to the variance of the single-subject principal component data, are subjected to Infomax ICA or SOBI, ultimately providing us with the time courses of the group components or source estimates. Finally, the subject-specific source time courses ${\tilde{s}}_{k}$ and the corresponding topographies ${\tilde{t}}_{k}$ were back-reconstructed.

The data simulations as well as the implementations of the four analytic approaches were scripted in MATLAB (R2012a, The MathWorks Inc., Natick, MA, 2000). Notable exceptions are the implementation of Infomax ICA, SOBI, as well as functions for data filtering and plotting of EEG topographies; for these tasks, routines of the MATLAB-based open source software package EEGLAB were used (Delorme and Makeig, 2004).

### 2.3. Performance measures and statistical assessment

Three main dependent variables were derived from the reconstructed single-subject sources for the evaluation of the algorithms: (1) correlations of frequency-spectra between the original and the reconstructed sources were computed and the highest correlation for each of the original sources was determined; (2) for each pair of an original and a reconstructed source as matched in (1), the correlation of amplitude variations was calculated; (3) the reconstruction accuracy, i.e., the variance of the original source explained by the variance of the reconstructed source, was computed based on the time domain representations of both the source and the matched independent components.

Regarding the first measure (spectral correlations), for the original and the reconstructed component time courses of a subject, 512 consecutive data points starting from the 1000 ms time stamp (presentation of the virtual stimulus) were extracted from every trial. These data excerpts were subjected to a discrete Fourier transform separately for each trial and subsequently averaged across trials. This yielded three frequency spectra for the original sources (alpha, beta, and gamma), and 62 frequency spectra of the components reconstructed for every subject. Then, correlations between the spectra of the original on the one, and the reconstructed components on the other hand were computed. From this correlation matrix, the highest correlation, averaged across subjects after Fisher-z transform, of any of the reconstructed components with each of the three original sources was extracted providing us with dependent variable 1.

Whereas measure 1 relies on frequency-domain representations of the signals, the computation of the remaining two measures is done in the time-domain. Dependent variable 2 assesses the quality of the reconstruction of amplitude variations at the single-trial level. To derive this measure, those three components identified during computation of measure 1, and each matching one of the original sources, were processed using a band-pass filter with a width of 8 Hz centered at the frequency of the matched source. Then, these data were rectified and temporally smoothed by using a 10-point lagged moving average: i.e., the value at time point *t* was computed as the average of the amplitude at time point *t* and its nine preceding data points. Subsequently, the maximum amplitude of each of the processed trials was computed and correlated with a vector coding the amplitude variations of the original source. Filtering and temporal smoothing were applied to compensate for the effects of noise, which was not present in the original source model but inevitably affects source estimation and reconstruction, thereby further differentiating it from the following measure.

The reconstruction accuracy (dependent variable 3) was computed to represent the percentage of the original source's variance in the time domain explained by the reconstructed independent component as identified for the computation of measure 1. More specifically, again 512 data points starting at 1000 ms were extracted from each trial, temporally concatenated, and the correlation of the original and the reconstructed time course was computed. When squaring the correlation coefficient, it indicates the percentage of mutually explained variance. Thus, in contrast to measure 2, variable 3 is well sensitive to the effects of noise; i.e., a reconstructed independent component capturing a substantial degree of noise may show high correlations regarding single-trial amplitude variations (measure 2), but low reconstruction accuracy due to an attenuated SNR.

Note that these measures are calculated on the single-subject data. However, in many cases the average correlation will be of interest. To compute the latter, correlation coefficients are first Fisher-z transformed, which represents a variance-stabilizing transformation, averaged, then computing the inverse Fisher-z transform of the average prior to reporting the results.

One measure that has recently been suggested for the evaluation of independent components is “dipolarity,” which basically relies on the match between the components' maps and the projection of single equivalent dipoles to scalp electrodes (Delorme et al., 2012). We did not apply this measure for our evaluations though, because we used simplified topographies with discrete loadings, which is not uncommon in simulation studies of EEG and fMRI. Real EEG topographies, however, are smoother, and sometimes more complex. Simplified topographies as applied in the context of this study, although fully sufficient for the evaluations applied here, are not well suited for inverse modeling, or dipole localization, as would be necessary for the dipolarity index as suggested by Delorme et al. (2012).

In sum, the design of this study corresponded to a four-factorial design of the simulations with factors SOURCE (alpha, beta, gamma), JITTER (0, 25, 50, 100, 200), TOPOGRAPHY (constant, variable), and ALGORITHM (tcICA, mlICA, tcSOBI, mlSOBI). NOISE conditions (Gaussian, EEG) were not directly statistically compared, because this would have violated some of the statistical assumptions of variance-analytic measures. ANOVAs corresponding to this design were computed for each of the three dependent variables and the two NOISE conditions separately. Although performance measures based on correlation values underwent Fisher-z transform prior to statistical assessment, figures will report the underlying correlation values to ease interpretation. Follow-up analyses relied on *post-hoc* computations with Tukey-tests. Statistical assessments were done using the Statistica software (10, Stat Soft Inc., Tulsa, OK, USA) and MATLAB (R2012a, The MathWorks Inc., Natrick, MA, 2000).

## 3. Results

### 3.1. Simulations with Gaussian noise

**Figure 4:**
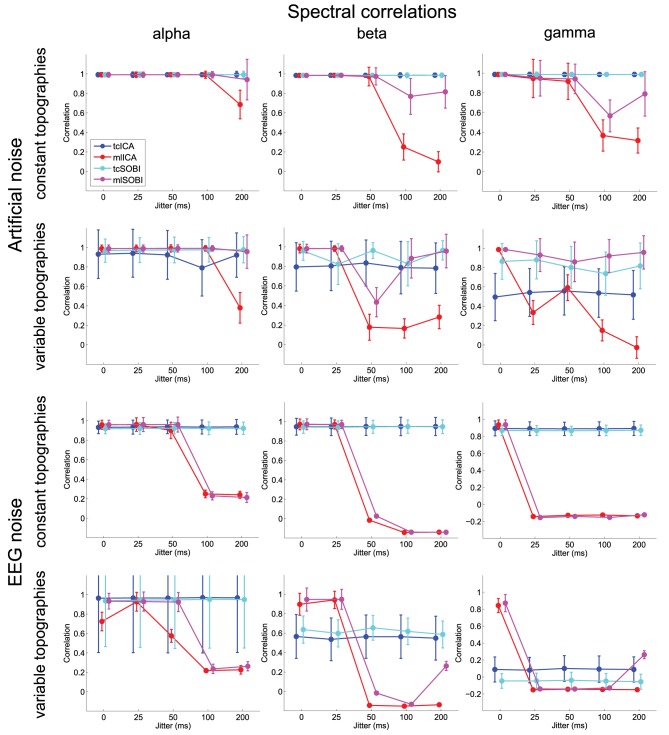
**Shown are means and standard deviations for spectral correlations between the simulated and reconstructed sources**. This measure provides feedback on the algorithms' ability to separate spectrally distinct sources.

**Figure 5:**
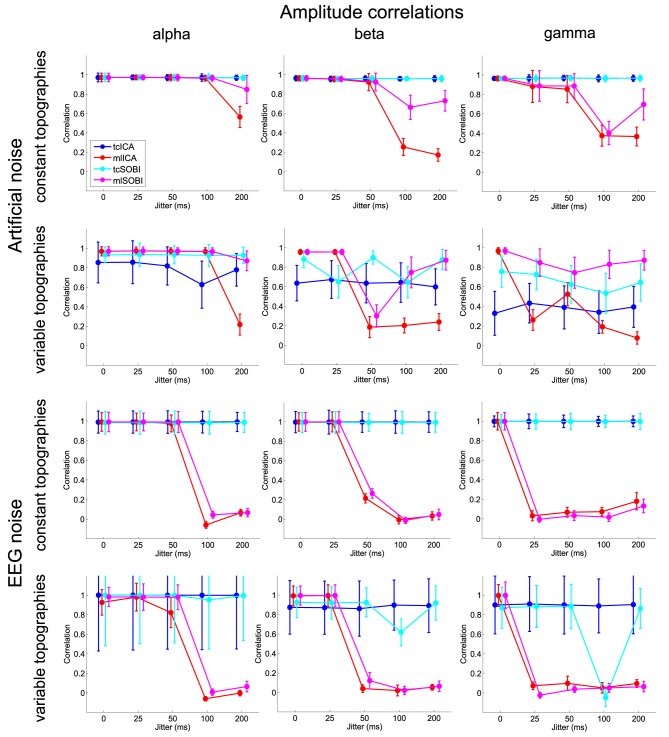
**Shown are means and standard deviations of the amplitude correlations, i.e., the correlation of the amplitude variability across trials as modeled in the simulated and captured in the reconstructed sources**.

**Figure 6:**
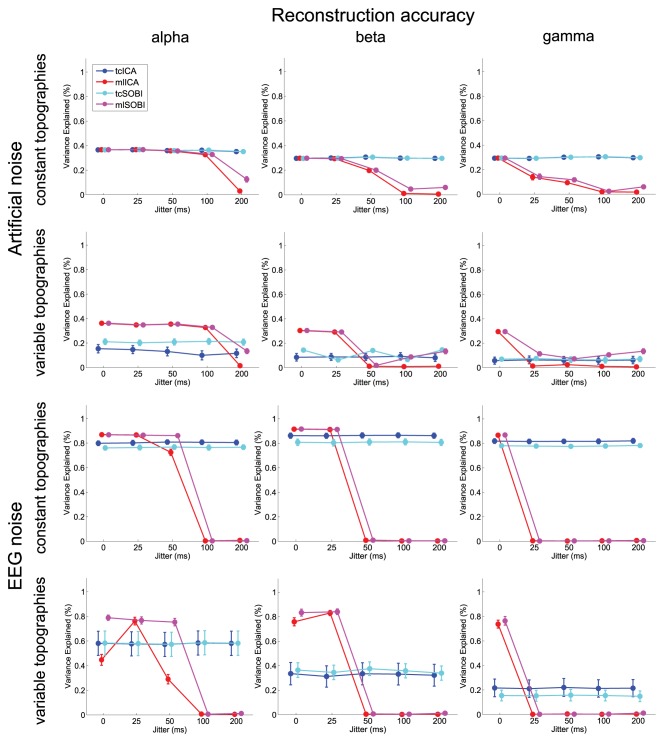
**Depicted are means and standard deviations of reconstruction accuracies, a measure that indicates the algorithms' capabilities to isolate the signal and attenuate the noise**.

Means and standard deviations, reported in accordance with the different combinations of the factor-levels, can be found for spectral correlations, amplitude correlations, and reconstruction accuracies in Figures 4–6, respectively.

#### 3.1.1. Effects of temporal jitter

As listed in Table 1, all three dependent variables revealed the expected two- and three-way interactions of ALGORITHM × JITTER and ALGORITHM × JITTER × SOURCE, as well as the four-factorial interaction including TOPOGRAPHY, to be significant. Irrespective of a source's frequency, with a temporal jitter of 200 ms, tcICA and tcSOBI outperformed mlICA in all measures (all *p* < 0.001). MlSOBI sometimes showed a performance in-between, but overall revealed a similar pattern as mlICA. More importantly though, the performance of mlICA and mlSOBI was modulated by an interaction of JITTER × SOURCE, which altogether was the cause of the aforementioned three-way interaction; the higher a source's frequency, the lower the temporal jitter necessary to deteriorate the performance of mlICA and mlSOBI. This effect was most clearly seen with reconstruction accuracy (see Figure 6), where alpha, beta, and gamma sources were associated with significant decreases in the explained variance at jitters of 200, 50, and 25 ms (all *p* < 0.001), respectively.

**Table 1:** **Summary of statistical effects computed from simulations using Gaussian noise**.

**Measure, Effects**	***F*-value**	**df, df_*error*_**	***p*-value**
**MAXIMUM SPECTRAL CORRELATION**			
ALGORITHM × JITTER	98.66	5.21, 72.99	< 0.001
ALGORITHM × JITTER × SOURCE	22.96	13.18, 80.03	< 0.001
ALGORITHM × TOPOGRAPHY	52.61	1.47, 20.63	< 0.001
ALGORITHM × TOPOGRAPHY × JITTER	11.15	4.33, 60.6	< 0.001
ALGORITHM × TOPOGRAPHY × JITTER × SOURCE	9.37	4.83, 81.04	< 0.001
**AMPLITUDE CORRELATION**			
ALGORITHM × JITTER	99.89	4.82, 67.4	< 0.001
ALGORITHM × JITTER × SOURCE	21.22	6.16, 85.89	< 0.001
ALGORITHM × TOPOGRAPHY	76.09	1.38, 19.29	< 0.001
ALGORITHM × TOPOGRAPHY × JITTER	11.51	2.38, 73.5	< 0.001
ALGORITHM × TOPOGRAPHY × JITTER × SOURCE	8.59	2.65, 86.59	< 0.001
**RECONSTRUCTION ACCURACY**			
ALGORITHM × JITTER	251.27	3.38, 47.25	< 0.001
ALGORITHM × JITTER × SOURCE	53.36	3.73, 52.28	< 0.001
ALGORITHM × TOPOGRAPHY	139.22	1.34, 18.8	< 0.001
ALGORITHM × TOPOGRAPHY × JITTER	11.71	3.86, 53.99	< 0.001
ALGORITHM × TOPOGRAPHY × JITTER × SOURCE	8.07	3.62, 50.67	< 0.001

Degrees of freedom (df) have been corrected according to Greenhous-Geisser for violation of sphericity; the same holds for the corresponding F- and p-values.

#### 3.1.2. Effects of varying mixing matrices

All three measures showed the expected two- and three-way interactions (ALGORITHM × TOPOGRAPHY, and ALGORITHM × TO-POGRAPHY × JITTER), as well as the four-factorial interaction including SOURCE. Test statistics are again listed in Table 1 and the effects are depicted in Figures 4–6. First, compared to constant mixing matrices, the performance of tcICA and tcSOBI significantly drops in all measures when the mixing matrices vary across subjects, an effect evident at all levels of JITTER (all *p* < 0.001). A similar, yet less pronounced effect, is seen with mlICA and mlSOBI. Importantly, the more pronounced drop in performance with tcICA and tcSOBI leads to significantly higher amplitude correlations and reconstruction accuracies with mlICA and mlSOBI in case of variable mixing matrices (all measures with *p* < 0.01).

JITTER, however, significantly modulates the associations of ALGORITHM and TOPOGRAPHY. Although the performance of mlICA, but not necessarily mlSOBI, drops with increasing temporal jitter with both conditions of TOPOGRAPHY, they also provide significantly better performance measures with jitters of 0, 25, or 50 ms (all *p* < 0.005), and significantly worse performance with a jitter of 200 ms (all three measures with *p* < 0.001). As can be seen in Figures 4–6, the exact performance pattern associated with JITTER also depends on the sources' frequency as already listed in the previous paragraph, altogether explaining the observed interaction including all four experimental factors.

## 3.2. Simulations with EEG noise

### 3.2.1. Effects of temporal jitter

Overall, the effects utilizing sources embedded in EEG noise show results comparable to those found with simulations using Gaussian noise. Again, both the two- and three-way interactions (ALGORITHM × JITTER and ALGORITHM × JITTER × SOURCE) turned out significant with all three dependent variables. Please refer to Table 2 for the test statistics. All measures show deteriorated performance with mlICA and mlSOBI as compared to tcICA and tcSOBI with temporal jitters of 100 ms and above (all *p* < 0.001); beyond, beta and gamma sources show even earlier onsets of deteriorated performance starting at jitters of 50 and 25 ms, respectively (all *p* < 0.05), giving rise to the aforementioned three-way interaction.

**Table 2:** **Summary of statistical effects computed from simulations using EEG noise**.

**Measure, Effects**	***F*-value**	**df, df_*error*_**	***p*-value**
**MAXIMUM SPECTRAL CORRELATION**			
ALGORITHM × JITTER	895.06	2.31, 32.35	< 0.001
ALGORITHM × JITTER × SOURCE	252.59	19.56, 61.53	< 0.001
ALGORITHM × TOPOGRAPHY	4.85	1.04, 14.58	< 0.001
ALGORITHM × TOPOGRAPHY × JITTER	34.44	3.25, 44.88	< 0.001
ALGORITHM × TOPOGRAPHY × JITTER × SOURCE	7.23	4.61, 64.52	< 0.001
**AMPLITUDE CORRELATION**			
ALGORITHM × JITTER	423.14	2.97, 41.51	< 0.001
ALGORITHM × JITTER × SOURCE	153.45	2.96, 41.46	< 0.001
ALGORITHM × TOPOGRAPHY	5.21	1.18, 16.53	< 0.04
ALGORITHM × TOPOGRAPHY × JITTER	42.24	2.49, 34.87	< 0.001
ALGORITHM × TOPOGRAPHY × JITTER × SOURCE	6.58	2.98, 41.74	< 0.002
**RCONSTRUCTION ACCURACY**			
ALGORITHM × JITTER	2331.17	2.61, 36.55	< 0.001
ALGORITHM × JITTER × SOURCE	685.9	4.76, 66.59	< 0.001
ALGORITHM × TOPOGRAPHY	43.53	1.13, 15.85	< 0.001
ALGORITHM × TOPOGRAPHY × JITTER	40.36	2.65, 37.09	< 0.001
ALGORITHM × TOPOGRAPHY × JITTER × SOURCE	22.22	4.74, 66.42	< 0.001

Degrees of freedom (df) have been corrected according to Greenhous-Geisser for violation of sphericity; the same holds for the corresponding F- and p-values.

However, the spectral correlation measure and the reconstruction accuracy show better performance with mlICA and mlSOBI and beta and gamma sources at jitter-levels up to 25 and 0 ms, respectively (all *p* < 0.02).

### 3.2.2. Effects of varying mixing matrices

Again, the two-way interaction of ALGORITHM × TOPOGRAPHY as well as the 3- and 4-way interactions were statistically significant with all three measures. Please refer to Table 2 and Figures 4–6 for statistics and depictions of the effects. Overall, all three dependent measures revealed a drop in performance with tcICA and tcSOBI from constant to variable topographies (all *p* < 0.05). As already noted, these findings were furthermore modulated by factors JITTER and SOURCE, such that mlICA and mlSOBI effectively showed better performance with specific combinations of temporal jitters and sources on the one, and variable topographies on the other hand. These effects were found with all dependent variables, but were most pronounced with the spectral correlations and reconstruction accuracies, especially at higher source frequencies. With beta and gamma sources, for example, performance of mlICA and mlSOBI was significantly better with variable as opposed to constant topographies at jitters of up to 25 and 0 ms, respectively (all *p* < 0.02). A similar outcome was found with the alpha source regarding mlSOBI, and partially also with respect to mlICA (e.g., Figure 6).

## 4. Discussion

The concurrent analysis of data sets obtained from multiple subjects for the estimation of latent variables representative for these aggregate data is a powerful tool that already has proven its usability in context of magnetic resonance imaging. With EEG though, the number of algorithms currently available is rather limited. The use of Infomax ICA in combination with either a temporal or vertical concatenation of data sets has been suggested. In addition, more recently SOBI has been brought forward as alternative to ICA for the processing of EEG (Lio and Boulinguez, 2013). However, altogether these early approaches have not yet fully considered major sources of variance in EEG recordings, namely topographic variability and differences in time-locking of neuronal responses across trials. With the present study our goal was to systematically evaluate and validate the four possible combinations resulting from the two procedures for data aggregation and the two prevalent source separation procedures. This way one could not only identify potential strengths and weaknesses of these methods, but also derive some guidelines for their application.

Overall, we found our hypotheses on the performance of these methods, when varying relevant characteristics of the simulated data sets, to be supported. When multi-level data decomposition is used (mlICA, mlSOBI; Eichele et al., 2011) a strong bias toward evoked activity patterns is found: the stronger the temporal jittering of source onset times across trials, the lower the performance. In addition, cancelation and decorrelation are more pronounced for a given degree of temporal jitter with high as compared to low frequency sources. As a consequence, tcICA and tcSOBI outperformed mlICA and mlSOBI with induced as compared to evoked source activity patterns. This pattern of results was very similar for both EEG and Gaussian background noise. Hence, this clearly supports the notion that using a group-level PCA as second preprocessing and data-reduction step on the aggregated multi-subject data does induce a strong bias toward evoked activity and lower frequency sources. Data reduction, however, is a necessary step because of the extremely high computational load associated with high-density EEG recordings, suggesting the need for further adaptations of this procedure.

On the other hand, we found the previously described performance pattern to be strongly affected by variations of source topographies, which could easily result from inconsistent electrode placement (minor effects) or inter-individual differences in neuroanatomy (major effects). That is, when topographies of the sources varied across subjects, the performance of the algorithms relying on temporal concatenation (Cong et al., 2013) significantly dropped, effectively leading to performance levels significantly lower than those of mlICA and mlSOBI at least with low degrees of temporal jittering. This effect was most pronounced with reconstruction accuracies, suggesting high degrees of noise relative to the signal when reconstructing source activities. Again, the effects were most pronounced with high frequent oscillatory activity, with performance decreases ranging from about 10 to 60% of the explained variance from alpha to gamma sources, respectively. Given the significant degree of variability of subject topographies observable across recordings, a very pronounced susceptibility for decreased performance with real EEG recordings is to be expected.

Previous work found that SOBI may well be suited to compensate for differences in electrode placement that usually occur across subjects during preparation of measurements (Lio and Boulinguez, 2013). In their work, Lio and Boulinguez used SOBI in combination with a temporal concatenation of multi-subject data. Consequently, one might hope that SOBI, and especially tcSOBI, would exhibit a better performance when subject topographies show a stronger degree of variability as would be caused by differences in neuroanatomy. Our findings, however, do only provide mixed support for this notion. Whereas with Gaussian background noise indeed all three measures indicated performance increases of mlSOBI relative to mlICA, this was not necessarily the case with sources embedded in EEG as background activity. Similar, but even less consistent, effects were found with tcSOBI and tcICA. Overall, it seems that SOBI, because of its reliance on lagged cross-correlations of input variables, may actually be more useful in compensating for temporal jitter than for topographical variability. Hence, future work needs to more systematically evaluate the relationship of the temporal lags used for SOBI's source estimation, the actual frequency of a source, as well as the degree of a source's time-locking.

The resuls clearly show that topographical variability of source projections can deteriorate the reconstruction of source time courses if not adequately compensated for. However, in this study we did not directly evaluate the accuracy of reconstructed source topographies. This is, because all procedures tested here allow for the reconstruction of single-subject source time courses, as this corresponds to the major strength of temporal ICA. However, only mlICA and mlSOBI so far also recover subject-specific source topographies. Source topographies recovered via tcICA or tcSOBI may substantially be distorted though, once topographical variability is present. Nonetheless, source topographies provide impportant information guiding the interpretation of underlying neurocognitive processes. Future work thus needs to refine procedures for the reconstruction of source topographies, as well as their evaluation. When assessing reconstruction accuracies of topographies, procedures could either be directly evulated in the electrode, as well as in the 3D source space when utilizing forward and inverse modeling (Delorme et al., 2012).

In addition, our evaluations support that characteristics of the inevitable measurement noise constitute critical factors for the algorithms' performance. This can, for example, clearly be seen in the lower performance measures with Gaussian as opposed to EEG noise. This effect may at first appear counterintuitive, yet is not fully unexpected since Gaussian noise may lead to suboptimal orthogonalization of the input variables (Arora et al., 2015). Indeed, the characteristics of noise may become even more relevant if procedures for the estimation of the model-order are utilized. When analyzing real EEG data, one might retrieve an estimate of the number of relevant sources by assessing the intrinsic dimensionality of the data (e.g., Camastra, 2003). Yet, current research suggests a strong dependence of the accuracy of such methods on the distributional characteristics of noise (e.g., Majeed and Avison, 2014). The effects of noise characteristics on both the methods for data decomposition and model-order estimation need further study though, such that no definite recommendation can be given yet on how to optimally configure a processing pipeline given different attributes of noise.

Based on the observations of this study, there is no general recommendation for one of the approaches, since none would be expected to perform well in all possible conditions. Temporal concatenation and subsequent source separation may be suggested as method of choice if a study's focus is on the estimation of sources generating induced responses. A thorough inspection of individual topographies needs to ensure that only minor differences between topographies of subjects exist, well accepting though that this inspection does not fully guarantee that the prerequisites of tcICA/tcSOBI are fully met, since a given topography can well be generated by different source constellations (known as the EEG inverse problem; e.g., Grech et al., 2008). However, should marked differences between subject topographies occur, or should the decomposition of evoked EEG activity be the major aim anyways, multilevel PCA in combination with ICA/SOBI would be a better choice. The latter framework could also be improved easily by sorting trials according to a criterion that would, at least partially, compensate for possible inter-trial variability in source onsets. When thinking about motor-related beta activity, for example, one could simply sort trials according to reaction times of responses and order the trials of subjects such that the inter-subject differences for all consecutive trials are minimized. It is obvious though that such criteria cannot always be derived easily. There is tasks, for example, that do not involve overt responding of subjects. Also, many brain processes may be recorded in the EEG, yet stay hidden because of strong overlap with other processes and the lack of an easily accessible outside criterion.

Procedures for group-level data decomposition and analyses offer powerful tools for the study of brain processes, not least because they allow for an easy statistical assessment of the identified sources, a feature not necessarily provided by *post-hoc* clustering of sources originally extracted for individuals. However, this study revealed the necessity to optimize current procedures to better address typical characteristics of multi-subject EEG data sets, namely inter-subject and inter-trial variability of topographies and source onset times, respectively. We are confident that this study will initiate further developments as well as refinements of the existing procedures, thereby contributing to the expansion of the tool set for the analysis of large-scale neuroscientific data.

### Conflict of interest statement

The authors declare that the research was conducted in the absence of any commercial or financial relationships that could be construed as a potential conflict of interest.

## Footnotes

^1^Further processing steps, such as z-transformations of input variables, are omitted for notational simplicity.
